# RUNX1-Evi-1 fusion gene inhibited differentiation and apoptosis in myelopoiesis: an in vivo study

**DOI:** 10.1186/s12885-015-1961-y

**Published:** 2015-12-16

**Authors:** Lijing Shen, Jianyi Zhu, Fangyuan Chen, Wenjie Lin, Jiayi Cai, Jihua Zhong, Hua Zhong

**Affiliations:** 1 Department of Hematology, Ren Ji Hospital, School of Medicine, Shanghai Jiao Tong University, Shanghai, 200127 China; 2 Department of Hematology, Ren Ji Hospital, School of Medicine, Shanghai Jiao Tong University, 160 Pujian Road, Shanghai, China

**Keywords:** RUNX1-Evi-1, Zebrafish, Myelopoiesis, Apoptosis, Valproic acid

## Abstract

**Background:**

Acute myeloid leukemia (AML) 1-Evi-1 is a chimeric gene generated by the t (3; 21) (q26; q22) translocation, which leads into malignant transformation of hematopoietic stem cells by unclear mechanisms. This in vivo study aimed to establish a stable line of zebrafish expressing the human RUNX1-Evi-1 fusion gene under the control of a heat stress-inducible bidirectional promoter, and investigate its roles in hematopoiesis and hematologic malignancies.

**Methods:**

We introduced human RUNX1-Evi-1 fusion gene into embryonic zebrafish through a heat-shock promoter to establish Tg(RE:HSE:EGFP) zebrafish. Two males and one female mosaic F0 zebrafish embryos (2.1 %) were identified as stable positive germline transgenic zebrafish.

**Results:**

The population of immature myeloid cells and hematopoietic blast cells were accumulated in peripheral blood and single cell suspension from kidney of adult Tg(RE:HSE:EGFP) zebrafish. RUNX1-Evi-1 presented an intensive influence on hematopoietic regulatory factors. Consequently, primitive hematopoiesis was enhanced by upregulation of gata2 and scl, while erythropoiesis was downregulated due to the suppression of gata1. Early stage of myelopoiesis was flourishing with the high expression of pu.1, but it was inhibited along with the low expression of mpo. Microarray analysis demonstrated that RUNX1-Evi-1 not only upregulated proteasome, cell cycle, glycolysis/gluconeogenesis, tyrosine metabolism, drug metabolism, and PPAR pathway, but also suppressed transforming growth factor β, Jak-STAT, DNA replication, mismatch repair, p53 pathway, JNK signaling pathway, and nucleotide excision repair. Interestingly, histone deacetylase 4 was significantly up-regulated. Factors in cell proliferation were obviously suppressed after 3-day treatment with histone deacetylase inhibitor, valproic acid. Accordingly, higher proportion of G1 arrest and apoptosis were manifested by the propidium iodide staining.

**Conclusion:**

RUNX1-Evi-1 may promote proliferation and apoptosis resistance of primitive hematopoietic cell, and inhibit the differentiation of myeloid cells with the synergy of different pathways and factors. VPA may be a promising choice in the molecular targeting therapy of RUNX1-Evi-1-related leukemia.

## Background

RUNX1-Ecotropic viral integration site (Evi)-1 chimeric gene is generated by the t(3;21)(q26;q22) translocation and plays a pivotal role in progression of different hematopoietic stem cell malignancies [myelodysplastic syndrome (MDS), chronic myelogenous leukemia to acute blastic crisis phase, and acute myelogenous leukemia (AML)]. RUNX1, also named AML1, is essential for hematopoietic cell development in fetal liver as well as lineage-specific differentiation in adult liver. Point mutations of RUNX1 are relatively common in M0AML (12-33 %), MDS (23 %), and therapy-related and radiation-associated MDS/AML (38-46 %) [1]. Evi-1 is a nuclear transcription factor that plays an essential role in the regulation of hematopoietic stem cells. Aberrant expression of Evi-1 has been reported in up to 10 % of patients with AML, which can predict poor outcome as a diagnostic marker [2]. However, bone marrow cells of murine transduced with Evi-1 alone cannot cause leukemia [3], while deletion of RUNX1 alone does not immortalize bone marrow cells [4], which suggest that both suppression of RUNX1 and activation of Evi-1 are required for RUNX1-Evi-1 leukemogenesis. It could be assumed that RUNX1-Evi-1 shares several molecular processes with wild-type Evi-1 and RUNX1 is more widely expressed in hematopoietic cells than Evi-1, it causes activation of Evi-1 function in hematopoietic cells, thus enhance cellular proliferation.

**Fig. 1:**
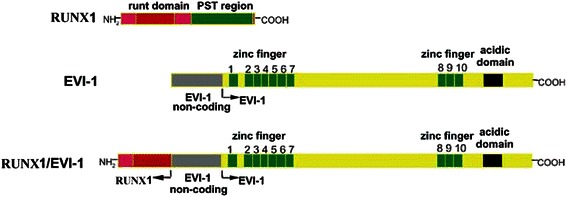
Schematic structure of wild-type RUNX1, Evi-1 and RUNX1/Evi-1 [30]. Wild-type RUNX1 possesses RHD at the N-terminus and PST region at the C-terminus. In RUNX1/Evi-1, N-terminal RUNX1 sequences are abruptly interrupted at the end of the RHD and followed by almost the entire coding region of Evi-1, including Evi-1 noncoding region, the first zinc finger domain, the second zinc finger domain, and an acidic (acidic amino acid-rich) domain are shown by boxes

Evi-1 protein has two separate Cys_2_His_2_-type zinc finger domains. In RUNX1-Evi-1 chimeric protein, the N-terminal half of RUNX1 including a runt homology domain (RHD) is fused to the entire zinc-finger Evi-1 (Fig. 1). Takeshita et al. demonstrated that the entire sequence of Evi-1 was required for transformation of primary bone marrow leukemia cells by RUNX1-Evi-1 [5]. Mice transplanted with RUNX1-Evi-1 retroviral transduced bone marrow cells suffered from an AML 5–13 mo after transplantation. The disease could be readily transferred into secondary recipients with a much shorter latency [6]. In another distinct BMT mouse model, Evi-1 seemed to collaborate with an RUNX1 mutant harboring a point mutation in the Runt homology domain (D171N) to induce with an identical phenotype characterized by marked hepatosplenomegaly, myeloid dysplasia, leukocytosis, and biphenotypic surface markers [7]. However, all of these sick mice died soon after transplantation, and generation of transgenic offspring to carry on the follow-up study is impossible.

Zebrafish (Danio rerio) hematopoiesis shows anatomic, physiologic, and genetic conservation with that of humans [8]. Furthermore, ectopic expression of human or murine oncogenes driven by specific promoters in zebrafish has been shown to faithfully develop leukemia closely parallel to the human leukemia subtypes [9]. Finally, the efficient reproduction and rapid development of zebrafish embryos allow it to become a convenient model to investigate tumor development and dissemination in real time even without sacrificing the animals.

In this study, we established a stable line of zebrafish expressing the human RUNX1-Evi-1 fusion gene under the control of a heat stress-inducible bidirectional promoter to examine its roles in hematopoiesis and hematologic malignancies. Interestingly, the phenotypes of these fish resembled to those of the human’s MDS-RAEB or AML. This transgenic strategy was based on previous studies [6, 10].

## Methods

This study was approved by the Institutional Animal Care and Use Committee (IACUC) of Shanghai Research Center for Model Organisms in China (approved ID: 2010–0010).

### Plasmid construction

**Fig. 2:**
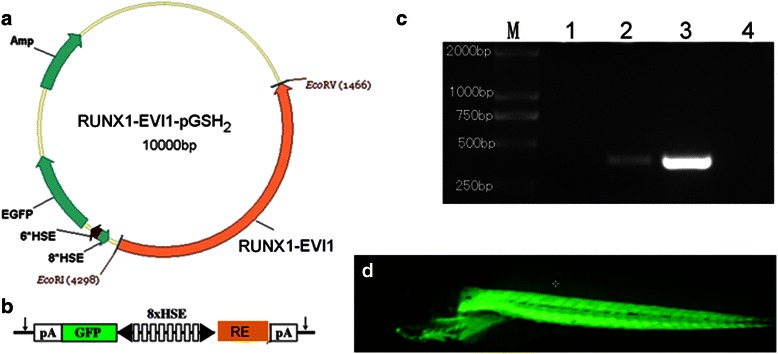
Generation of Tg(RE:HSE:EGFP) zebrafish line. (**a**) Schematic diagram of the structure of PSGH2/RUNX1-Evi-1 recombinant plasmid. A human-RUNX1-Evi-1 fragment was cloned into the EcoRI and EcoRV sites of the PSGH2 vector. (**b**) A schematic presentation of the eight multimerized heat shock element (HSE) promoter, which is flanked by two minimal promoters in opposed orientation (black arrowhead) to bidirectionally induce EGFP and RUNX1-Evi-1 expression. The vector is flanked by I-SceI meganuclease sites (arrows). pA, SV40 polyadenylation signal. (**c**) Transgenic verification by PCR: M: TAKARA DL2000 marker; lane 1 and 2: wild type and Tg(RE:HSE:EGFP) zebrafish larvae at 3 dpf, respectively; lane 3: PSGH2/RUNX1-Evi-1 plasmid; lane 4: double distilled water. (**d**) EGFP expression in Tg(RE:HSE:EGFP) zebrafish F2 generation at 3dpf (×4)

The cDNA of human AML1(RUNX1)-Evi-1 was identified and obtained from the SKH-1 cell line and inserted into the EcoRI site of pME18S, named as pME-AE (generous gift from Motohi Ichikawa) [5]. It was subcloned into EcoRI and EcoRV (Takara, Japan) sites of the pSGH2 vector [10], which contains eight HSE sequence (AGAACGTTCTAGAAC) and EGFP segment. Then, we obtain the hRUNX1-Evi-1-HSE-EGFP insert construct (Fig. 2a), HSE allows the symmetrical addition of a CMV minimal promoter to both ends in order to drive the expression of two interested genes (EGFP at one side and hRUNX1-Evi-1 at the other side) flanked by 5 V and 3 V globin UTRs and SV40 polyadenylation (pA) signal (I-SceI meganuclease recognition sites) (Fig. 2b).

### Generation of the Tg(RE:HSE:EGFP) zebrafish line

Zebrafish was maintained as described by Westerfield [11]. Developmental stages refer to hours or days post fertilization (hpf or dpf). Fertilized wild type (WT) AB fish eggs were microinjected through the chorion into the cytoplasm at the one-cell stage of development according to our previous work [12]. The pSGH2-hRUNX1-Evi-1 plasmid was co-injected with I-SceI meganuclease enzyme (0.5 units/μL) (New England Bio Labs). A pressure injector (IM-300, NARISHIGE) was used with borosilicate glass capillaries. After injection, the embryos were collected in Petri dishes and incubated at 28 °C. Heat shock was executed at 38 °C for 1 hour at between 14 to 18 hpf to induce EGFP and RUNX1-Evi-1 expression. EGFP+ fish were screened under the fluorescent microscope on the next day and bred up to sexual maturity, then crossed with the WT AB fish. The transgenetic (Tg) offspring also received heat shock for an hour to induce the target genes expression.

### Real-time quantitative reverse transcription PCR (qRT-PCR) and western blot analysis

QRT-PCR was performed as described previously [11]. It was performed using 400 ng of cDNA templates in an ABI StepOnePlus System (Applied Biosystems, USA). PCR primers were designed to span introns and listed in Table 1. Measured cycle threshold (Ct) values represent log2 expression levels. Each target gene was normalized to β–actin and calculated using the 2-ΔΔCT method [13]. Deyolking embryos and protein immunoblotting were performed as described [14]. Anti-JNK (No.9252) and anti-actin antibodies were purchased from Cell Signaling Technology (Beverly, MA). Anti-p-JNK (No.sc-12882) was purchased from Santa Cruz Biotech (USA).

**Table 1:** RT-PCR Primers

Name	Forward primer	Reverse primer	Product (bp)
RUNX1-Evi-1	ATATCGCTGCGAAGACTGTGACCA	TGAAGGTTGCTAGGGTCCGTGAAA	381
scl	GAACAGTATGGGATGTATCCTAGC	CGTTGAGGAGCTTAGCCAGA	265
lmo2	ACACTGGAGGCAAATGAGGAG	AGTAAAGCCTGCGTCCCACC	193
gata2	CTGCCAGACAACCACGACC	CCAGATCGTTTACTCCTCTTGG	180
gata1	CCATCGTATTATTCCACCAGC	GGATGTGGGGTTGTAGGGAG	159
pu.1	TCCCAGCAGTCGTAGTCCTC	CCATTTCGCAGAAGGTCAA	141
mpo	GGGTTGACCACGATCTGACT	CAGGGAGACAGGTGTTAGGG	159
c-myb	GGCAGAAAGTCCTCAACCC	ATCGGTTTCCAAGTTTCTCG	252
runx1	TGGGACGCCAAATACGAAC	AGGACGGAGCAGAGGAAGTT	227
P21	GAACGATGTGCTGCACTCCC	TGTCAATAACGCTGCTACGAGAC	236
P27	CGACTGTAGGGTAACGGAGCA	GGGTGTCGGACTCAATGGTT	178
P53	CGGCGATCATGGATTTAGGC	TTCAGCCACATGCTCGGACT	202
ras	GTCCACGATGAATCCCGAATA	TCTCCTGCCCTGCTGTATCC	212
Bax	ACAGGGATGCTGAAGTGACC	GAAAAGCGCCACAACTCTTC	236
Bcl2	AACGCGAATTTGAGGAAATG	TATCTACCTGGGACGCCATC	189
JNK	AACAGGAATAAGCGCGAGAA	TTGTGGTTGACGCATTTCAT	245
JUN	AAACAGCGCTTTCTCTCAGC	TGATCATGCCGTTGCTAGAC	221
skp2	ATCTGGGACTGAGCCGTTGT	GAGAACGGCTGCGTGTTGAT	166
Caspas3	AGGCTTGTCGAGGAACAGAA	CATGATCTGCAAGAGCTCCA	233
smad7	GGTTCTGTGCCTGCTTCCA	TGCCCTGAGGTAGGTCGTAGA	202
smad4	AGACCTCCACATACCACCACA	GTCCATCTCGAAGTAGGCAAT	221
β-actin	CCTGACCGAGAGAGGCTACA	CGCAAGATTCCATACCCAAG	242

### *In situ* hybridization

Whole-mount *in situ* hybridization was performed with digoxigenin-labeled (Roche) antisense riboprobes for hematopoietic transcription factors (scl, lmo2, gata1, pu.1, mpo) according to our previous work [15].

### Cytological analysis

After transferred into 50 mg/L tricaine for 1 ~ 3 min, blood was harvested from zebrafish by making a lateral incision just posterior to the dorsal fin in the dorsal aorta area and used in preparing blood smears [16]. Slides were then stained with Wright Giemsa stain and examined under oil immersion by light microscopy. Identification of zebrafish peripheral blood cells was based, in part, on previous descriptions of teleost blood cells [17]. Single cell suspensions of kidney were collected and filtered by 40 μm mesh, than stained by the same method.

### Microarray analysis

The WT and Tg(RE:HSE:EGFP) F2 generation embryos were heated shocked at 38 °C for 1 hour at 16 hpf, then raised to 3 dpf. Total RNAs were isolated with Trizol (Invitrogen). The samples were processed and subsequently analyzed in triplicate on Zebrafish Oligo Microarrays (Agilent Technologies Italia, Italy) which contain 43,554 sets of probes. The microarrays were scanned in an Agilent DNA Microarray Scanner and the images were processed using Feature Extraction software. Functional annotation analysis was performed using NIH-DAVID software (version 6.7) to find the most relevant Kyoto Encyclopedia of Genes and Genomes (KEGG) terms associated with differentially expressed genes (DEGs) [18]. For this purpose, the significance p-value threshold was set as <0.01, with Bonferroni multiple testing correction (<0.01).

### Drug administration

Valproic acid (VPA, CAS Number: 1069-66-5, Sigma-Aldrich Co. LLC., USA) was dissolved with DMSO and then diluted into different concentrations of 5, 10, 25, 50, 100, 250, 500 μM (DMSO < 0.2 % in each to avoid the toxicity of DMSO [19]) in egg water. 30 embryos were maintained in individual wells in 12-well microtitre plates at 28 °C from 14 hpf. At 24 and 48 hr after VPA treatment, larvae were collected for LD50 confirmation and gene screen.

### FACS profile analysis

Tg(RE:HSE:EGFP) larvae were crushed and cell suspensions were homogenized in ice-cold 0.9× phosphate-buffered saline (PBS) containing 5 % fetal bovine serum, and then passed through a 40 μm filter to obtain a single cell suspension. These single cells treated with red blood cell lysis solution and washed once with PBS, stained with Propidium Iodide (Sigma-Aldrich) at a final concentration of 1 μg/mL and analyzed by fluorescence-activated cell sorting (BD FACS ARIA II SORP, USA) to investigate apoptosis in RUNX1-Evi-1 positive cells.

### Statistical analysis

Data were analyzed on GraphPad Prism 5 using one-way ANOVA and unpaired Student’s t test. Differences were considered significant at p values of less than 0.05.

## Results

### Establishment of Tg(RE:HSE:EGFP) zebrafish line

About 40 % of the embryos injected with the pSGH2-RUNX1-Evi-1 plasmid exhibited EGFP+ expression after heat shock at 38°Cfor an hour. The adult EGFP+ fish was crossed with the WT fish. The Tg(RE:HSE:EGFP) F0 founders with the highest germline transmission rate were identified on the basis of fin genotyping (Fig. 2c-d) and EGFP expression of the F1 offspring after the same heat shock treatment. Three of 146 (2.1 %) mosaic F0 zebrafish were identified as the stable germline Tg zebrafish, including 2 males and 1 female. The Tg F1 generation were mated to create homozygous Tg(RE:HSE:EGFP) line. The EGFP+ frequency of F2 offspring reached to 75 % after heat shocked.

### RUNX1-Evi-1 induced immature hematopoietic cells emerged in blood circulation

**Fig. 3:**
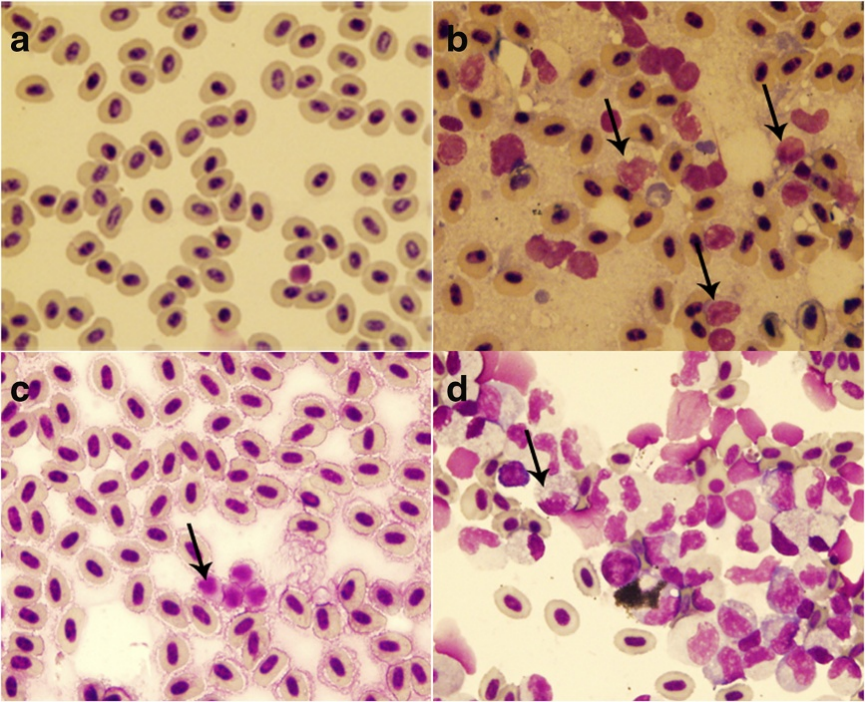
Cytological analysis of Tg(RE:HSE:EGFP) zebrafish. Cytology of hematopoietic cells from WT (**a**) and Tg(RE:HSE:EGFP) F2 generation (**b**) zebrafish at 60 dpf. The blood cells from WT fish were predominantly erythrocytes, and by contrast, erythrocytes were significantly inhibited in Tg(RE:HSE:EGFP) fish, enriched for abundant blast-like cells, which are larger than the erythrocytes and have high nuclear to cytoplasmic ratios, containing multiple large nucleoli (black arrow). These blasts were similar to that of human AML peripheral blood. The similar feature presented in single cell suspensions of kidneys from WT (**c**) and Tg F2 generation (**d**)

Using Wright Giemsa staining, peripheral blood from WT zebrafish at 60 dpf contained clusters of erythrocytes, while myeloid cells were only occasionally observed (Fig. 3a). In contrast, the blood cells from the Tg(RE:HSE:EGFP) fish contained some blast-like cells, which were larger than the erythrocytes and had high nuclear to cytoplasmic ratios with multiple large nucleoli. These cells resembled to human AML blasts. Meanwhile, erythrocytes were significantly inhibited (Fig. 3b). The similar feature presented in single cell suspensions of kidney from WT and Tg F2 generation (Fig. 2c-d).

### RUNX1-Evi-1 reprogrammed lineage-specific hematopoietic transcription factors

**Fig. 4:**
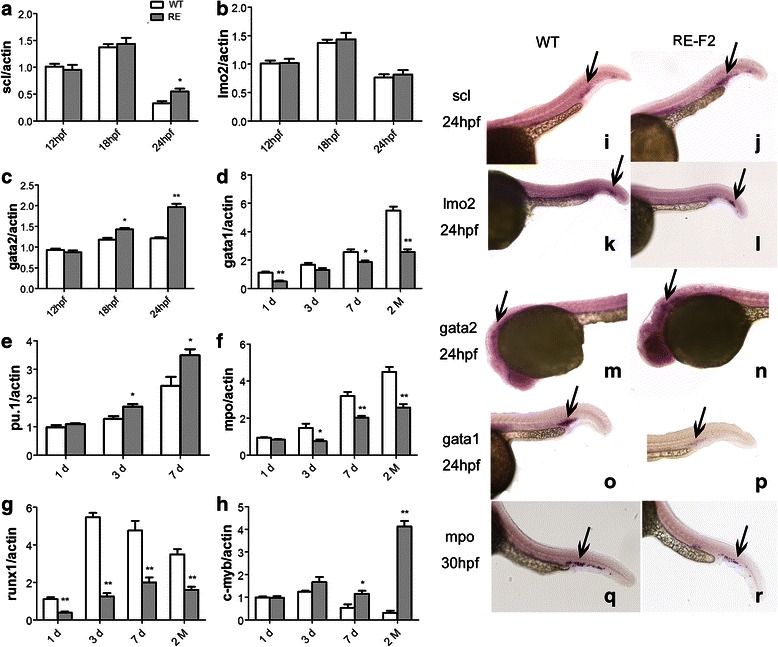
RUNX1-Evi-1 reprogrammed lineage-specific hematopoietic transcription factors. Scl (**a**), lmo2 (**b**) and gata2 (**c**) were detected by qRT-PCR in WT, and Tg(RE:HSE:EGFP) F2 generation embryos at 12 hpf, 18 hpf and 24 hpf. Gata1 (**d**), pu.1 (**e**), mpo (**f**), runx1(**g**), and c-myb (**h**) expressed in WT, Tg F2 zebrafish at 1 dpf, 3 dpf, 7 dpf and 60 dpf. Compared with WT, scl, gata2, pu.1, and c-myb were up-regulated, while gata1, mpo, and runx1 were down-regulated in Tg fish. *In situ* hybridization of scl (**i**-**j**), lmo2 (**k**-**l**), gata2 (**m**-**n**), and gata1 (**o**-**p**) at 24hpf and mpo (**q**-**r**) at 30hpf in WT and Tg F2 embryos demonstrated the same tendency. *P < 0.05; **P < 0.01

Similar to mammalian, zebrafish also experienced a primitive wave and a definitive wave of hematopoiesis. From the 2 somite stage, cells co-expressing stem cells transcription factor (scl), gata2 and LIM only protein 2 (lmo2) transcription factors bilaterally appeared in both the anterior lateral mesoderm (ALM) and the posterior lateral mesoderm (PLM). They have the potential to become HSCs, but not kidney progenitors [20]. The expression of scl, lmo2, and gata2 were measured by qRT-PCR and *in situ* hybridization in WT and Tg(RE:HSE:EGFP) F2 generation embryos at 12hpf, 18hpf and 24 hpf. Scl was slight higher expression in Tg embryos than that in WT at 24hpf (0.329 ± 0.066 *vs* 0.547 ± 0.096, P = 0.032) (Fig. 4a, i-j). No significant difference of lmo2 expression between WT and Tg(RE:HSE:EGFP) zebrafish was identified (Fig. 4b, k-l). Gata2 was upregulated compared to WT counterpart at 18 hpf (1.180 ± 0.075 *vs* 1.426 ± 0.066, P = 0.013) and 24 hpf (1.211 ± 0.045 *vs* 1.965 ± 0.144, P = 0.001) (Fig. 4c, m-n).

**Fig. 5:**
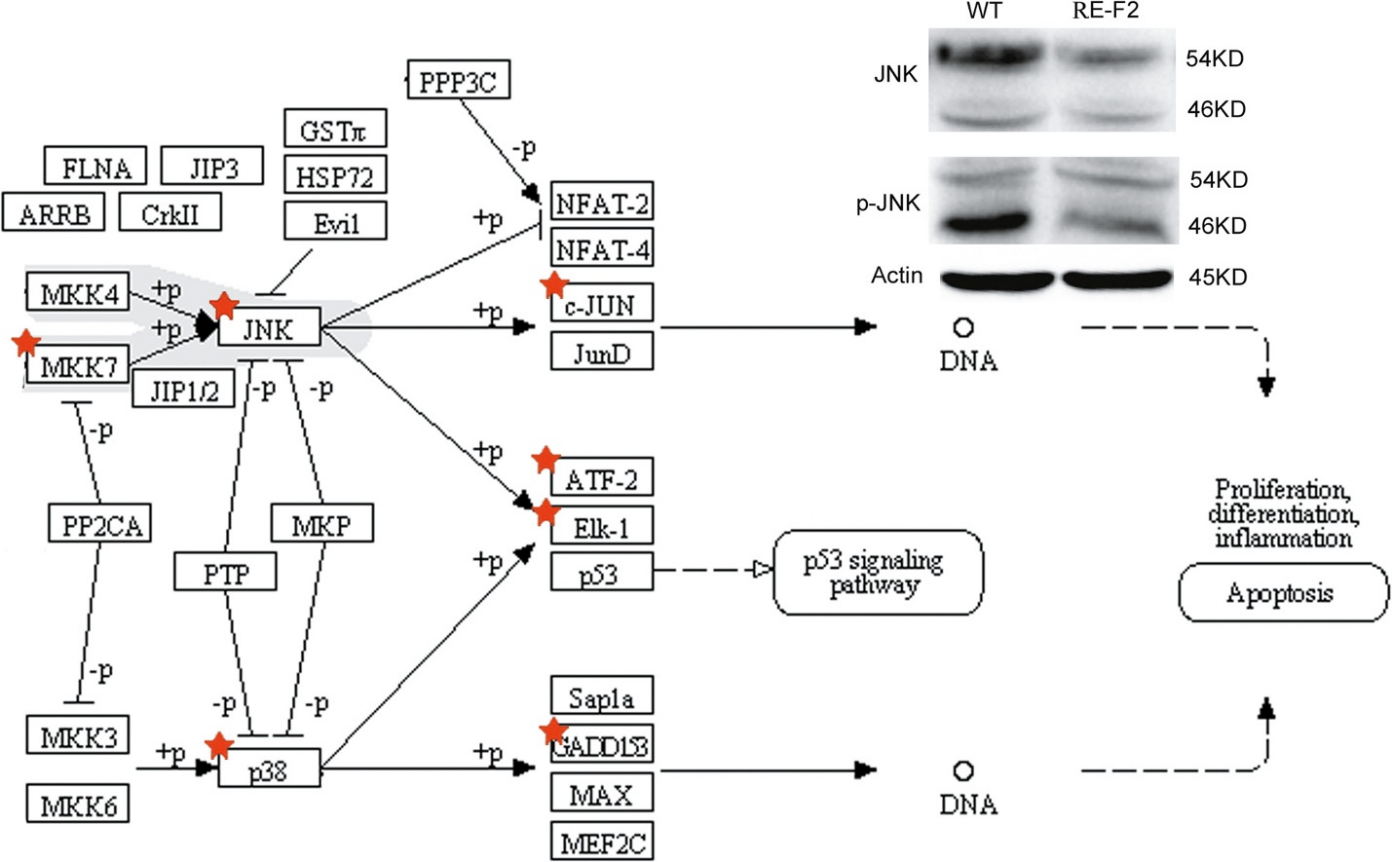
DEGs involved in JNK pathway in Tg(RE:HSE:EGFP). Using Agilent microarray analysis, compared with WT at 3dpf, it showed that RUNX1-Evi-1 could down-regulate some crucial genes in JNK pathway, including c-JUN, ATF-2, Elk-1, and GADD153. MKK7 is a functional gene in MAPK pathway, which was also down-regulated in this model. The signaling pathways were analyzed and summarized by NIH-DAVID software (in KEGG) and the differentially expressed genes were marked with red star in the automatically generated figure. Western blot was further confirmed that JNK and cleaved p-JNK expression were inhibited in Tg(RE:HSE:EGFP) F2 generation

Gata1 and pu.1 is the master regulator in erythrocyte and myeloid cell development involved in primitive hematopoiesis, respectively. In zebrafish, gata1 is expressed from the 5 somite stage in the PLM. According to the results in WT and Tg embryos at 1 dpf (1.114 ± 0.126 *vs* 0.493 ± 0.097), 3 dpf (1.674 ± 0.237 *vs* 1.308 ± 0.236), 7 dpf (2.565 ± 0.321 *vs* 1.863 ± 0.192), and 60 dpf (5.496 ± 0.470 *vs* 2.573 ± 0.328). It was demonstrated that the levels of gata1 significantly decreased in Tg embryos (Fig. 4d, o-p). Pu.1 is expressed from the 6 somite stage in the ALM. Compared with WT, its expression was upregulated in Tg groups at 3 dpf (1.274 ± 0.165 *vs* 1.702 ± 0.155) and 7 dpf (2.432 ± 0.540 *vs* 3.496 ± 0.367) (Fig. 4e).

Like in mammals, the first definitive HSCs of zebrafish arise from the ventral region of the dorsal aorta, and express runx1 and c-myb transcription factors. Unlike primitive HSCs, definitive HSCs have the potential to become all blood lineages including lymphocytes. Runx1 were dramatically downregulated in Tg(RE:HSE:EGFP) embryos at 1 dpf (1.114 ± 0.156 *vs* 0.393 ± 0.099), 3 dpf (5.474 ± 0.402 *vs* 1.256 ± 0.296), 7 dpf (4.765 ± 0.892 *vs* 1.996 ± 0.470), and 60 dpf (3.496 ± 0.470 *vs* 1.609 ± 0.274) (Fig. 4g). Whereas, the expression of c-myb, whose expression is predominantly present in immature hematopoietic cells and decreases during cell differentiation, did not decrease with cell growth and differentiation in Tg fish at 60 dpf (0.314 ± 0.158 *vs* 4.122 ± 0.419, P = 0.000) (Fig. 4h). It was indicative of a large number of immature blood cells accumulating in blood circulation.

As the downstream gene of pu.1, myeloperoxidase (mpo) was the granulocyte specific gene and considered as the symbol of mature neutrophils, whose expression was firstly detected in between 18 and 20 hpf with the distribution from the intermediate cell mass (ICM) to rostral blood island (RBI). In Tg fish, the mpo expression obviously reduced at 3 dpf (1.474 ± 0.402 *vs* 0.753 ± 0.158), 7 dpf (3.199 ± 0.373 *vs* 2.029 ± 0.173), and 60 dpf (4.496 ± 0.470 *vs* 2.576 ± 0.325) (Fig. 4f, q-r). It’s not enough to support the myeloid cells’ further development and differentiation.

### RUNX1-Evi-1 changed multiple transcriptional pathways in transgenic larvae

Using Agilent microarray analysis, we obtained a total of 578 DEGs in the blood cells of Tg(RE:HSE:EGFP) F2 generation *vs* WT larvae at 3dpf. There were 348 genes upregulated and 230 genes downregulated (2-fold change in expression, *P* < 0.01). Analyzed by NIH-DAVID software, several KEGG pathways were significantly enriched (*P* < 0.01, Benjamini < 0.01) (Table 2) (GSE74944).

**Table 2:** Change of signaling pathways in Tg(RE:HSE:EGFP) zebrafish

Term	Regulate	Count^a^	%^b^	*P*-Value	Benjamini
Proteasome	+	11	1.3	2.6E-9	2.8E-7
Cell cycle	+	28	2.2	5.3E-8	3.7E-6
Glycolysis/Gluconeogenesis	+	16	1.0	8.5E-6	7.0E-5
Tyrosine metabolism	+	10	0.9	9.1E-6	3.9E-5
Tryptophan metabolism	+	8	0.9	1.6E-5	4.1E-4
Drug metabolism	+	7	0.8	5.1E-5	7.6E-3
Metabolism of xenobiotics by cytochrome P450	+	7	0.8	5.1E-3	7.6E-2
Androgen and estrogen metabolism	+	8	0.9	7.0E-3	8.0E-2
PPAR signaling pathway	+	6	0.7	7.2E-3	7.4E-2
Retinol metabolism	+	4	0.5	8.4E-3	7.5E-2
Jak-STAT signaling pathway	-	14	2.4	6.8E-10	8.3E-8
DNA replication	-	15	1.1	3.5E-9	3.0E-7
Mismatch repair	-	8	0.9	4.5E-7	1.8E-6
Homologous recombination	-	12	0.9	8.9E-7	3.5E-5
Base excision repair	-	12	1	2.7E-6	4.7E-5
Spliceosome	-	22	1.6	2.9E-5	4.4E-4
Nucleotide excision repair	-	18	1.2	6.3E-5	4.3E-4
p53 signaling pathway	-	16	1.3	3.8E-4	8.3E-3
JNK signaling pathway	-	7	1.2	4.0E-4	8.7E-3
Butanoate metabolism	-	4	0.7	4.1E-4	2.7E-3
Transforming growth factor β	-	14	1.1	6.3 E-4	7.3 E-3
Porphyrin and chlorophyll metabolism	-	4	0.7	7.4E-4	7.8E-3
Arginine and proline metabolism	-	5	0.9	7.8E-4	8.0E-3

+: upregulated; −: downregulated. ^a^: genes involved in the term. ^b^: involved genes/total genes

*P*-Value: the threshold of EASE Score, a modified Fisher Exact P-Value, for gene-enrichment analysis (<0.01). Benjamini: Benjamini and Hochberg’s false discovery rate (<0.01)

Some signaling pathways were upregulated in the Tg(RE:HSE:EGFP) fish, including proteasome, cell cycle, glycolysis/gluconeogenesis, tyrosine metabolism, tryptophan metabolism, metabolism of xenobiotics by cytochrome P450, PPAR signaling pathway, etc. Glycolysis was often depended on for ATP production in rapidly proliferating tumors even in normoxia, which is defined as the Warburg effect. Here, RUNX1-Evi-1 also significantly upregulated the genes that correlated with glycolysis/gluconeogenesis (n = 16, P = 8.5E-6), including fructose-1, 6-bisphosphatase I, glucose-6-phosphate 1-epimerase, and glucose-6-phosphate isomerase.

Meanwhile, transforming growth factor β (TGFβ), Jak-STAT, DNA replication, mismatch repair, p53 pathway, JUN N-terminal kinase (JNK) signaling pathway, nucleotide excision repair were downregulated. JNKs belong to the superfamily of mitogen-activated protein kinases that are involved in the regulation of cell proliferation, differentiation and apoptosis [21]. Compared with WT, some crucial genes were downregulated in Tg samples (n = 7, P = 6.8E-4, Fig. 5). The proteins of JNKs and p-JNKs (the activated state) dramatically decreased in Tg(RE:HSE:EGFP) F2 larvae (Fig. 5).

Majority of the above pathway alterations were associated with human hematopoietic disorders and malignant transformation of blood cells [22–26]. These data needs to be verified in further experiments.

### VPA partially rescued some pathways abnormally regulated by RUNX1-Evi-1

Mitani found that RUNX1-related chimeras generated by the chromosomal translocations repress transcriptional activity of wild-type RUNX1 (AML1) by recruiting the co-repressor/histone deacetylase complex in leukemia cell lines. Further, as a histone deacetylase inhibitors(HDACi), VPA could elicit apoptosis through both extrinsic and intrinsic pathways in these cells [27]. Here, microarray analysis also showed that histone deacetylase 4 (hdac4) was significantly upregulated in Tg(RE:HSE:EGFP) F2 generation larvae (6.18 *vs* 10.62, fold change: 21.70, showed in Table 3). Therefore, we investigated the in vivo effects of VPA on Tg(RE:HSE:EGFP) zebrafish.

**Table 3:** RUNX1-Evi-1 upregulated histone deacetylase in microarray analysis

Genes	WT	AE-F2	FC	Regulation
Histone deacetylase 11 (HD11)	5.47	8.16	6.49	+
Histone deacetylase 4 (hdac4)	6.18	10.62	21.70	+
H1 histone family, member X (h1fx)	10.02	11.36	2.53	+

FC: fold change; +: upregulated

Tg(RE:HSE:EGFP) embryos were treated with different concentrations of VPA at 28 °C from 14 hpf after one hour of heat shock treatment. According to the outcome at 72hpf [Y = 29.18ln(x) + 0.49 (0.9799)], we got the LD50 as 69.72 μM, and chose 35 μM, 70 μM to carry on detecting the effectiveness. By FCM analysis, elevated apoptosis was presented in VPA treated groups, which was indicated by sub G1 peak. Compared with the untreated group, treated with 70 μM VPA induced apoptosis at (60.43 ± 7.28) % and decreased S/G2 cell population at [(87.37 ± 4.06) % *vs* (23.64 ± 3.23) %, *P* < 0.01], correspondingly, G1 arrest was increased.

Furthermore, we measured the expression of some crucial factors involved in proliferation and apoptosis pathways after treated with different concentrations of VPA by qRT-PCR (Fig. 7). It showed that VPA reduced the expression of skp2 and upregulated p21, p27, which would inhibit the progress of cell proliferation. Smad7 was also inhibited by VPA and revived the TGF β signaling. VPA significantly downregulated Bcl2, but enhanced the expression of P53, Bax, JNK and JUN, which indicated the activation of both extrinsic and intrinsic apoptosis. Majority change of these genes was concentration dependence (Fig. 7). In contrast, the expression of the above genes showed no significant difference in wild type zebrafish embryos treated with the similar dose of VPA (data not shown).

**Fig. 6:**
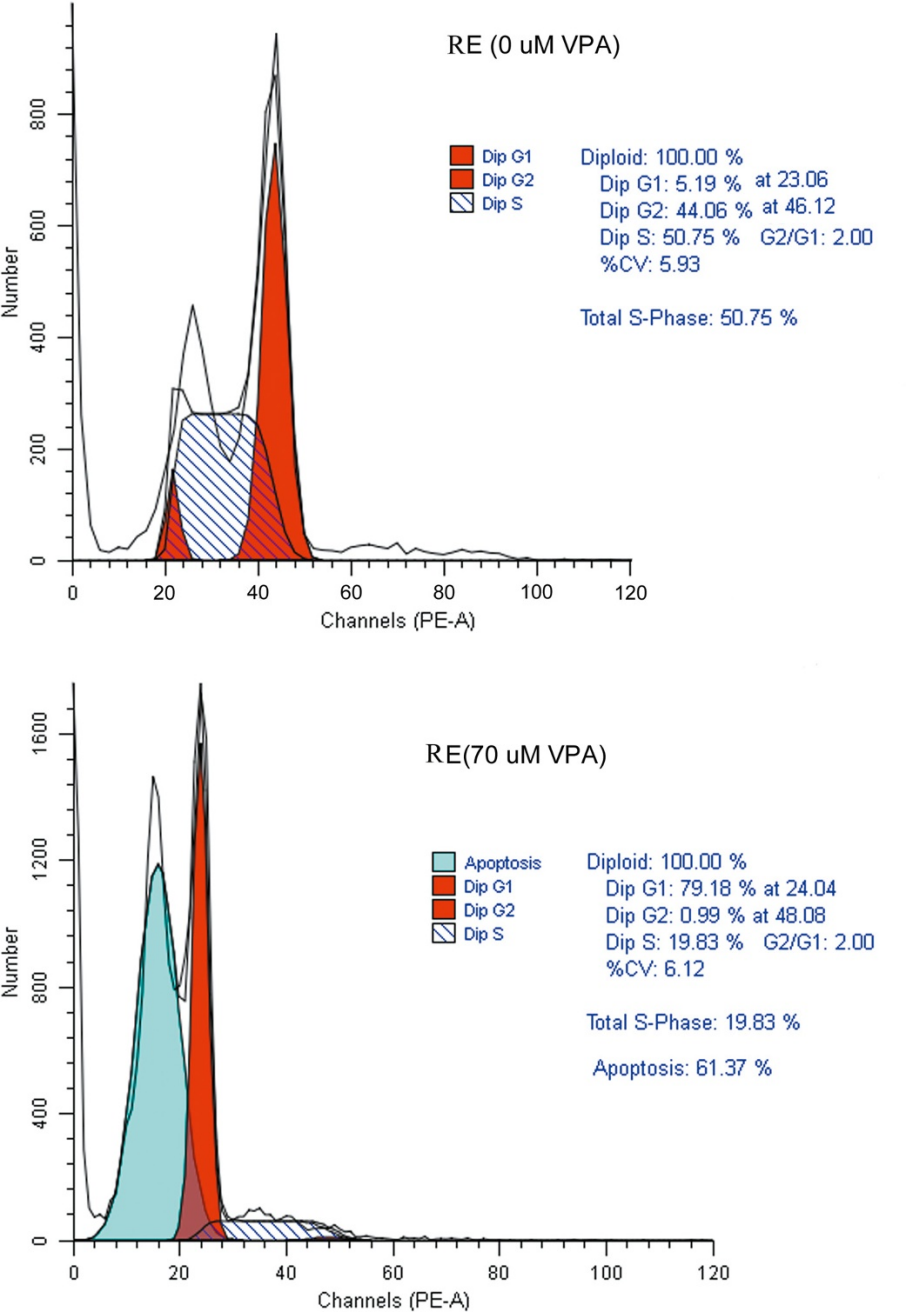
VPA promoted the G1 arrest and apoptosis in RUNX1-Evi-1 positive cells. The cells of Tg(RE:HSE:EGFP) embryos were sorted with GFP after two days treated by 70 μM VPA. The ratio of S/G2 phase was sharply decreased (94.81 % *vs* 20.82 %), accompanied with a sub-G1 peak (61.37 %)

### Followed-up of the transgenic zebrafish

Different from 3 years lifetime of the WT zebrafish, all these three F0 founders died within 8–14 months (median: 10.6 months). Most of the F1 and F2 generation gradually lost the EGFP expression and the ability of fecundity, and began to die older than 12 months. Along with the growth of Tg(RE:HSE:EGFP) zebrafish, some of them presented pathological appearance (e.g. hemorrhage, edema and small size) (Fig. 8).

**Fig. 7:**
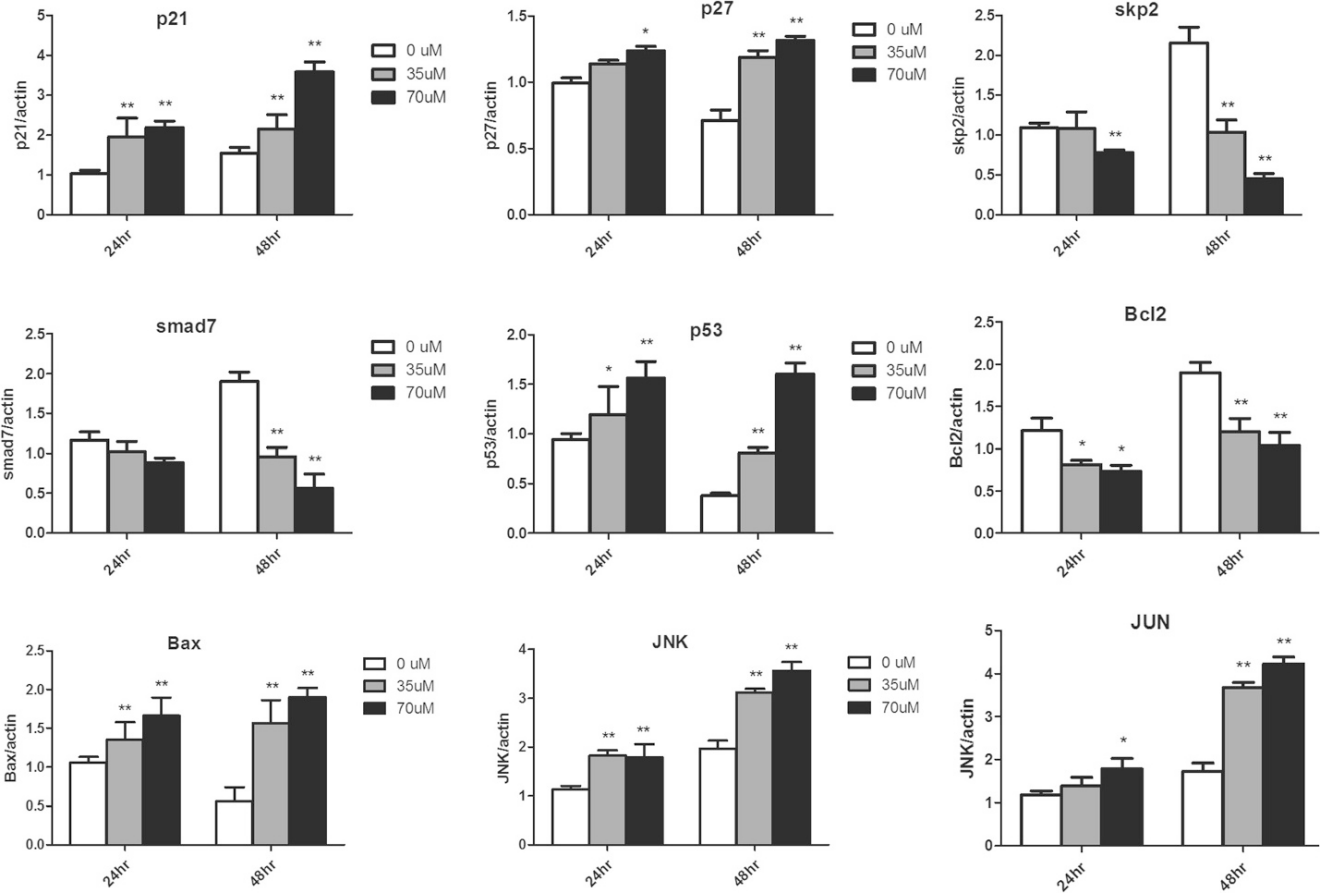
VPA changed some genes expression in Tg(RE:HSE:EGFP) embryos. VPA reduced the expression of skp2 and smad7, and upregulated p21 and p27, which inhibited the progress of cell proliferation. VPA significantly downregulated Bcl2, and meanwhile enhanced the expression of P53, Bax, JNK and JUN. Majority of these changes were concentration dependence. *P <0.05; **P < 0.01

## Discussion

Growing evidences demonstrated that normal function loss of Runx1/AML1 or abnormal activation of Evi-1 was an indicator of poor prognosis in leukemia [27–29]. It was also shared that RUNX1-Evi-1 fusion gene could enhance the malignant effectiveness [30]. Nevertheless, the major mechanism of RUNX1-Evi-1 exerts in acute leukemia and whether it possesses the whole role of Evi-1 remained widely unknown. We reported here an RUNX1-Evi-1 transgenic zebrafish model with a phenotype that recapitulated main aspects of human AML such as distorted proliferation, anti-apoptosis, anemia, increased immature myeloid cells and their precursors accumulated in peripheral circulation and kidney marrow (kidney marrow serves throughout the life of a zebrafish, generating adult hematopoietic cells, just like human’s bone marrow [20]), which suggested that RUNX1-Evi-1 played a role in the etiology of AML. More importantly, zebrafish offers the advantage of high-throughput scale in the study of RUNX1-Evi-1 function and drug screen in vivo, which enables us to track the molecular alterations that occur well before the appearance of morphological phenotypes, and to determine the roles of candidate RUNX1-Evi-1 target genes.

HSP has no tissue-specific preference, yet heat stress exhibits more direct and far-reaching influence on white blood cells than other cells. Moreover, RUNX1-Evi-1 overexpression is highly oncogenic in myeloid cells. Thus, the establishment of RUNX1-Evi-1 transgenic zebrafish with the uniform phenotype of the tumor cells shows better resemblance to the feature of human MDS/AML.

Carolyn [28] found disruption of terminal myeloid differentiation and cell cycle regulation to be prominent in Evi-1-induced leukemogenesis. Using microarray analysis, we also found the upregulation of cell cycle (n = 28, P = 5.3E-8) and repression of TGF-β signaling (n = 14, P = 6.3 E-4) in Tg(RE:HSE:EGFP) zebrafish, accompanied by increased expression of some early hematopoiesis transcription factors (gata2 and pu.1) (Fig. 4c, e, m-n). Gata2 was a zinc finger transcription factor which was required for proliferation and maintenance of hematopoietic progenitor cells [20]. Yuasa [31] showed that Evi-1 promoted early hematopoietic development in the P-Sp region, which seemed to depend on activation of gata2 and repression of TGF-β signaling, while ZF1 of Evi-1 directly recognized and banded to the gata2 promoter. Gata2 has been reported to be aberrantly expressed in 87 % of de novo AML cases while well correlation between Evi-1 and gata2 expression were found in AML patients [28, 32]. Thus, it’s feasible that gata2 plays a crucial role in RUNX1-Evi-1 or Evi-1-induced leukemogenesis. Likewise, Evi-1 interacted with pu.1 and repressed the pu.1-dependent activation of a myeloid promoter [21]. Leopoldo [21] reported on a mouse model that constitutive expression of Evi-1 in the BM led to fatal anemia and myeloid dysplasia, and Evi-1 interaction with gata1 blocks proper erythropoiesis. Here, RUNX1-Evi-1 slightly enhanced the expression of pu.1, but repressed the level of mpo, the downstream regulator of pu.1 (Fig. 4e-f, q-r). Meanwhile, gata1, the indispensable promoter for erythropoiesis, was overwhelmingly inhibited along with growth (Fig. 4d, o-p). Actually, there was a cross-inhibitory mechanism between the expression of gata1 and pu.1 in zebrafish [20], which indicated that the level of pu.1 expression was determined by the ability of RUNX1-Evi-1 to regulate both pu.1 and gata1, thus increased the repopulation of immature myeloid cells and anemia.

Evi-1 was a zinc finger-containing and site specific DNA-binding transcription factor, but it did not seem to inhibit pu.1 binding to DNA, but rather to block its association with the co-activator JNK and its substrates [21]. In response to cell death stimuli, p-JNKs activated apoptotic signaling by regulating apoptotic-associated genes via the transcriptional activation of specific transcription factors or by directly modulating the activities of mitochondrial apoptosis-associated proteins through distinct phosphorylation events [24]. In our previous work, we demonstrated that arsenic trioxide (ATO) could reactivate apoptosis in the THP1 (with high level of Evi-1 expression) cell line by downregulating Evi-1. ATO significantly decreased Bcl-2 and Bcl-xL expression, thus accordingly increased the levels of JNK, p-JNK, Bax, full length caspase-3 and cleaved caspase-3 in western blot analysis [33]. In the present study, RUNX1-Evi-1 fusion gene inhibited both JNK signaling and P53 pathway (Table 2). Using FCM, we found that more than 50 % of RUNX1-Evi-1 positive cells were in the S/G2 period and no sub-G1 peak (Fig. 6), which demonstrate apoptosis resistant and weak efficacy of G1 arrest. Because of the deficiency of enough monoclonal antibody for zebrafish and lethal effectiveness of RUNX1-Evi-1 to mouse embryos [34], we plan to do further study on the efficacy of ATO in Tg(RE:HSE:EGFP) zebrafish and confirm molecular mechanism in detail through retroviral transduction of RUNX1-Evi-1 into Evi-1 negative cell lines or adult mice.

**Fig. 8:**
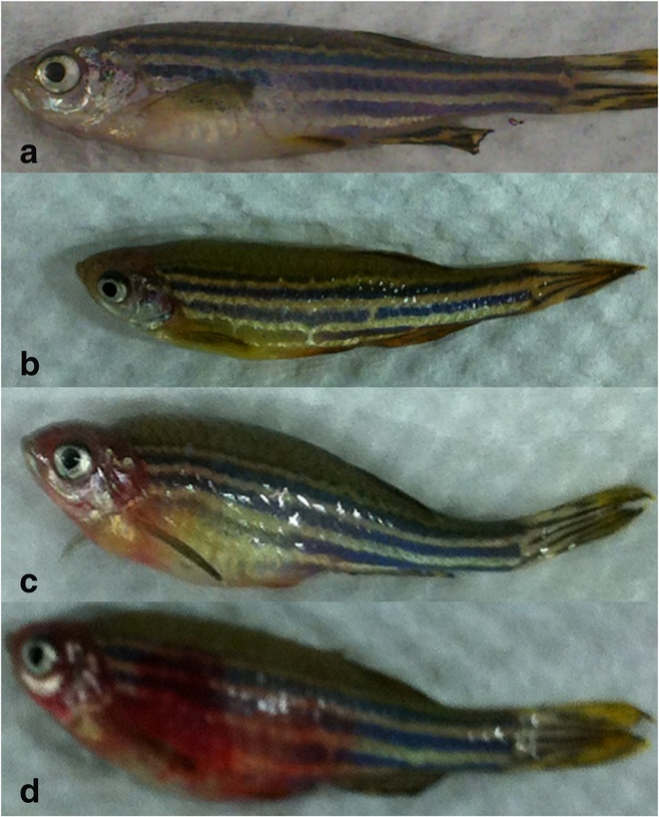
The appearance changes in Tg(RE:HSE:EGFP) adult zebrafish. (**a**) WT AB strain zebrafish; (**b-d**) various appearances with pathological changes presented in transgenic zebrafish, developmental delay (**b**), edema (**c**), and bleeding (**d**)

RUNX1-related chimeras generated by the chromosomal translocations repressed transcriptional activity of wild-type RUNX1 by recruiting the co-repressor/histone deacetylase complex [27]. Thus, histone deacetylase inhibitors were expected to restore normal functions of wild-type RUNX1, and thereby affect the growth and differentiation ability of leukemic cells expressing the chimera. Histone deacetylase 4 obiously increased in Tg(RE:HSE:EGFP) larvae (Table 3). Therefore, we investigated the in vivo effects of histone deacetylase inhibitor (VPA). G1 arrest and apoptosis progression were significantly enhanced in RUNX1-Evi-1 positive cells after 2-day treated with VPA (Fig. 6). Up- and downregulation of cell cycle and apoptosis regulator genes appeared to be the molecular basis for the former (Fig. 7). Therefore, VPA may be an attractive choice in the molecular targeting therapy of RUNX1-Evi-1-related leukemia.

60 years ago, some scholars have presented that disordered amino acid metabolic processes in patients with acute leukemia, such as eighteen patients with acute leukemia averaged 137.1 mg. of urinary “tyrosyl” in 24 hr. as compared to 115.8 mg. for normal individuals of the same age group [35]. Tyrosine kinase inhibitors (TKIs) therapy in leukemia cells creates a novel metabolic state that is highly sensitive to particular mitochondrial perturbations [36]. The essential amino acid L-tryptophan (L-TRP) is required for the biosynthesis of proteins and is precursor for several biologically important compounds, and the inhibition level of L-TRP metabolites in blood could be a useful parameter of chemotherapy efficacy for Adult T-cell Leukemia/Lymphoma patients [37]. In contrary, the majority of newly diagnosed AML patients’ blasts have deficiencies in the arginine-recycling pathway enzymes argininosuccinate synthase and ornithine transcarbamylase, making them arginine auxotrophic [38]. Miraki-Moud F et al. showed that AML cells from most patients with AML are deficient in a critical enzyme required for arginine synthesis, arginino succinate synthetase-1 (ASS1). Thus, these ASS1-deficient AML cells are dependent on importing extracellular arginine [39]. Using Agilent microarray analysis, we also found that tyrosine metabolism and tryptophan metabolism pathway were upregulated, while Arginine and proline metabolism were inhibited in the Tg(RE:HSE:EGFP) F2 generation. It suggested that the strategy of enzymatic degradation of amino acids to deprive malignant cells of important nutrients would be an established component of induction therapy of AML.

Interestingly, Maki [34] knocked-in the RUNX1-Evi-1 chimeric gene into mouse embryo, which led to defective hematopoiesis in the fetal liver and death around embryonic day 13.5 (E13.5) as a result of hemorrhage in the central nervous system. Maki hypothesized that maintained expression of pu.1 gene and decreased expression of lmo2 and scl genes may explain the aberrant hematopoiesis. We also found that some of the Tg(RE:HSE:EGFP) zebrafish died of hemorrhage (Fig. 8d). However, the levels of scl and lmo2 expression were similar to those in WT counterpart and the average lifespan was nearly 12 months. Taking the advantage of transparence in zebrafish embryo, we performed whole-mount in situ hybridization to confirm the difference expression of these factors, which were consistent with the results of qRT-PCR. The different outcome between Maki’s and ours may be associated with the different species, which needs further explore.

## Conclusion

Taken together, the phenotypes of Tg(RE:HSE:EGFP) fish resemble to those of the human’s MDS-RAEB or AML. RUNX1-Evi-1 promoted the primitive hematopoietic cell proliferation and apoptosis resistant, inhibited myeloid cells differentiation through synergy of several pathway and factors. This model provides a useful tool to conduct whole-organism chemical suppressor screens to identify compounds that can regain RUNX1 function and reverse Evi-1 in vivo. We propose VPA to be an attractive choice in the molecular targeting therapy of RUNX1-Evi-1-related leukemia.

## Abbreviations

AML: Acute myeloid leukemia
Evi: Ecotropic viral integration site
MDS: myelodysplastic syndrome
RHD: runt homology domain
IACUC: Institutional Animal Care and Use Committee
pA: polyadenylation
hpf or dpf: hours or days post fertilization
WT: wild type
Tg: transgenic
qRT-PCR: Real-time quantitative reverse transcription PCR
Ct: cycle threshold
KEGG: Kyoto Encyclopedia of Genes and Genomes
DEGs: differentially expressed genes
PBS: phosphate-buffered saline
ALM: anterior lateral mesoderm
PLM: posterior lateral mesoderm
scl: stem cells transcription factor
lmo2: LIM only protein 2
mpo: myeloperoxidase
ICM: intermediate cell mass
RBI: rostral blood island
TGFβ: transforming growth factor β
JNK: JUN N-terminal kinase
hdac4: histone deacetylase 4
VPA: valproic acid
ATO: arsenic trioxide
